# How educational innovations and attention to competencies in postgraduate medical education relate to preparedness for practice: the key role of the learning environment

**DOI:** 10.1007/s40037-015-0219-3

**Published:** 2015-10-26

**Authors:** Ids S. Dijkstra, Jan Pols, Pine Remmelts, Eric F. Rietzschel, Janke Cohen-Schotanus, Paul L.P. Brand

**Affiliations:** 1 Wenckebach Institute, University Medical Center Groningen, University of Groningen, FC 10, Hanzeplein 1, 9700 RB Groningen, The Netherlands; 2 Faculty of Behavioural and Social Sciences, University of Groningen, Groningen, The Netherlands; 3 Center for Research and Innovation in Medical Education, University of Groningen, University Medical Center Groningen, Groningen, The Netherlands; 4 Wenckebach Institute, University of Groningen, University Medical Center Groningen, and Princess Amalia Children’s Centre, Isala Hospital, Zwolle, The Netherlands

**Keywords:** Competency-based education, Evaluation, Learning environment, CanMEDS

## Abstract

**Introduction:**

Many training programmes in postgraduate medical education (PGME) have introduced competency frameworks, but the effects of this change on preparedness for practice are unknown. Therefore, we explored how elements of competency-based programmes in PGME (educational innovations, attention to competencies and learning environment) were related to perceived preparedness for practice among new consultants.

**Methods:**

A questionnaire was distributed among 330 new consultants. Respondents rated how well their PGME training programme prepared them for practice, the extent to which educational innovations (portfolio, Mini-CEX) were implemented, and how much attention was paid to CanMEDS competencies during feedback and coaching, and they answered questions on the learning environment and general self-efficacy. Multiple regression and mediation analyses were used to analyze data.

**Results:**

The response rate was 43 % (143/330). Controlling for self-efficacy and gender, the learning environment was the strongest predictor of preparedness for practice (B = 0.42, *p* < 0.001), followed by attention to competencies (B = 0.29, *p* < 0.01). Educational innovations were not directly related to preparedness for practice. The overall model explained 52 % of the variance in preparedness for practice. Attention to competencies mediated the relationship between educational innovations and preparedness for practice. This mediation became stronger at higher learning environment values.

**Conclusions:**

The learning environment plays a key role in determining the degree to which competency-based PGME prepares trainees for independent practice.

## Introduction

In many Western countries competency-based education (CBE) has been introduced in postgraduate medical education (PGME) to prepare trainees better for the growing complexities of the medical profession [1, 2]. However, some authors have challenged the rationale behind CBE and questioned whether the investment of effort, time and money required to incorporate CBE will achieve its promise [3–5]. Therefore, we set out to examine the relationship between CBE and its objective of better preparing physicians for independent practice.

Comparative studies of the outcomes of different curricula generally do not yield meaningful results because of the complexity of the intervention, the impossibility of blinding participants, and the difficulty in defining pure outcomes [6–9]. As a more promising alternative, theory-driven correlational studies have been proposed which endeavour to include all influential variables and interactions, rather than losing them through randomization [8]. In the present study we aim to explore the relationship between key elements of competency-based PGME and preparedness for practice using a conceptual model based on educational theory and previous research in the field, which we will elaborate on below.

## Development of conceptual research model

Along with CBE, various educational innovations have been introduced into PGME, such as portfolios, critically appraised topics and mini-clinical evaluation exercises. The use of portfolios enables the monitoring of a trainee’s progress through the curriculum, and helps to explicitly broaden the scope of evaluation and hence provide a way to longitudinally assess broad educational and professional outcomes [10, 11]. Moreover, the provision of structured and constructive feedback after observation of the trainee performing a task in practice (mini-clinical evaluation exercises) promotes dedicated attention to the various relevant competency domains [2, 11]. Because these innovations encourage both supervisors and trainees to consider and discuss improvements in all areas of competence, we expect educational innovations to be positively related to attention to competencies during feedback and coaching.

Competency frameworks were developed to broaden the scope of education beyond the medical expert role, for instance to encompass the roles of communicator, collaborator and manager [1]. The specification of these competencies allows trainees to comprehend the demands of medical training and medical practice [12]. As feedback is central to learning and has a positive effect on clinical performance [13, 14], trainees from programmes that pay closer attention to the various competency roles during feedback and coaching can be expected to feel better prepared for the challenges of contemporary medical practice. Accordingly, we expect attention to the domains of competence during feedback and coaching to be positively related to preparedness for practice. Because we expect a positive relationship between educational innovations and attention to competencies, and given the expected positive relationship between attention to competencies and preparedness for practice, we also expect a positive relationship between educational innovations and preparedness for practice, but we expect this relationship to be mediated by attention to competencies.

Educational innovations and attention to competencies only represent a part of all the factors that prepare trainees for practice during PGME. To make sound inferences, other equally important factors have to be taken into account, which could confound the relationship between these aspects of PGME modernization and preparedness for practice. Because PGME predominantly consists of work-based learning, a strong and supportive learning environment encompassing many important aspects—including quality of supervision, teaching, facilities and atmosphere [15]—is likely to provide more opportunities for experiential learning, more support to reflect on and learn from these experiences, and more opportunities to apply the various competency roles. Accordingly, we expect that the strength of the learning environment is not only positively related to preparedness for practice but we also expect it to moderate the relationship between attention to competencies and preparedness for practice.

**Fig. 1:**
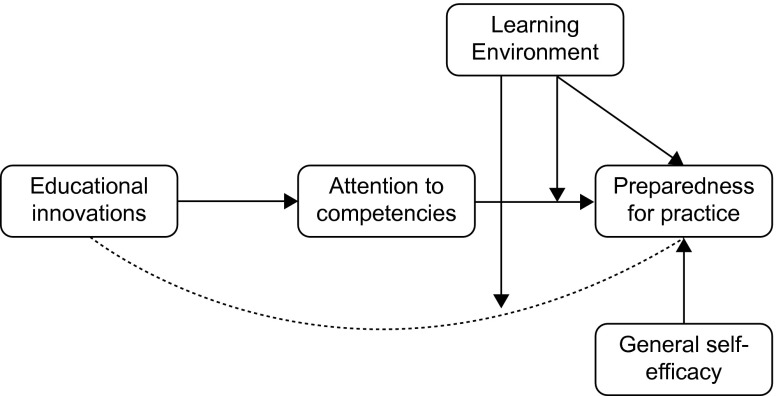
Research model: factors of PGME influencing preparedness for practice of new consultants

Finally, whether someone feels prepared for practice will also depend on the inherent qualities and mind-set of the individual. According to social cognitive theory, perceived self-efficacy is a predominant factor that influences behaviour [16]. Individuals high in general self-efficacy consistently view themselves as capable of meeting demands in multiple environments [17], which is why we expect self-efficacy to be positively related to preparedness for practice. Taken together, the aforementioned expected relationships form our conceptual research model, which is depicted in Fig. 1.

## Methods

### Context and participants

In the Netherlands, competency-based PGME training was introduced into the various PGME curricula from 2004 onwards. For the purpose of this study, PGME programme directors from all specialities in the northeast educational region of the Netherlands provided the contact details of former trainees who completed their training between 2004 and 2010.

In 2011, all respondents received an initial email in which the purpose and procedure of the study was announced. In this email all applicable ethical considerations were addressed. Respondents were informed about the voluntary nature of participation, confidentiality, anonymous processing and storage of all collected data and the possibility to withdraw their participation during any phase of the study. Accordingly, the study was in compliance with the declaration of Helsinki, established ethical standards [18, 19], and the legal requirements in the Netherlands. The initial email in which the study was announced was followed by a separate email with a link to a web-based questionnaire. Reminders were sent after 2, 4 and 8 weeks.

### Measures

Preparedness for practice was measured using a generic inventory of 91 medical specialist tasks [20]. Respondents were asked to rate how well their training programmes had prepared them for these tasks. More specifically, respondents were asked to respond to the statement ‘*my postgraduate training programme prepared me well for…* [Task]’ on a 5-point Likert scale ranging from 1 (strongly disagree) to 5 (strongly agree), or to indicate that they had not encountered such a task in their practice.

Since perceived preparedness for practice may be influenced by general confidence in the ability and capacity to accomplish a task, the Schwarzer and Jerusalem general self-efficacy scale was included as a control variable to balance its effect across subjects [21]. The questionnaire consists of 10 items scored on a 4-point Likert scale ranging from 1 (strongly disagree) to 4 (strongly agree). (*e.g. When I am confronted with a problem, I can usually find several solutions*).

The CanMEDS framework is used for PGME in the Netherlands [1]. The attention to the 7 CanMEDS competencies was assessed by asking participants to rate their agreement to 1 statement for every competency role (7 items): ‘*During my postgraduate training programme, attention was consistently paid to the competency…* [e.g. Professionalism] *in the course of coaching and feedback.*’ Answers could be rated on a 5-point Likert scale ranging from 1 (strongly disagree) to 5 (strongly agree).

In 2004, the first Dutch PGME programmes became competency-based. From 2011 onwards, CBE became mandatory for all PGME programmes, of which the requirements were recorded in the 2011 national guidelines for Dutch PGME programmes [22]. Using these guidelines, the authors discussed with residents, programme leaders and educational consultants which of these requirements were typical for the changes that had occurred in Dutch PGME, and differed fundamentally from the requirements in earlier guidelines. This resulted in a list of 6 educational innovations (Table 1). The questionnaire asked respondents to rate the use of these 6 innovations on a Likert scale as described above (e.g. *During my postgraduate training programme… I received feedback based on mini clinical evaluation exercises*).

**Tab. 1:** Items used to measure educational innovations and learning environment

**Educational innovations**
During my training, I formulated learning objectives for the different stages of my training, together with my programme director
During my training, I kept a portfolio
During my training, the content of my training was in accordance with the applying curricular documents
During my training, I received feedback based on mini clinical evaluation exercises
During my training, I made short summaries of a few scientific papers concerning a topic from clinical practice (CAT)
During my training, my knowledge was assessed by means of progress tests
**Learning environment**
During my training, the tasks and activities I had to perform grew along with my own development
During my training, I was able to independently perform all relevant aspects of my profession
During my training, there were opportunities to observe my supervisor and other consultants
During my training, there were opportunities to exchange experiences with other residents
During my training, there was scheduled time to increase my knowledge through reading of professional literature
During my training, I was allowed time to reflect on the tasks and activities I had performed
During my training, at completion of every rotation, we examined whether predefined learning objectives had been reached
During my training, I received feedback in a constructive way
During my training, my supervisors were readily available for advice and supervision
During my training, I had a good relationship with my supervisors and staff members
During my training, there was a good working atmosphere
During my training, there was clarity regarding my role

Several questionnaires have been developed to capture the concept of learning climates [23, 24]. These questionnaires generally contain many items and considering the amount of questions that our questionnaire already contained, we chose not to use these surveys. Alternatively we relied on Teunissen et al. [25] who developed a theoretical framework of resident work-related learning. According to their model, work-related learning essentially thrives on participating in activities, reflecting on them and subsequently adjusting new behaviour based on this reflection. Their model further incorporates the significance of social interaction and codified knowledge as driving forces of the learning cycle and acknowledges the organizational environment. We used these concepts for the development of our questionnaire and reasoned that a better integration of these concepts in training programmes is related to better learning outcomes. The items of our measure were held against the theoretical framework of learning environments as developed by Shönrock et al. [26] and neatly covered the domains of effective learning environments (goal orientation, relationships and organization). Respondents were asked to rate their agreement to 12 items on Likert scales (*e.g. During my postgraduate training programme… I was able to perform all relevant aspects of my profession independently*) (Table 1).

### Data analysis

Prior to analysis, means for all continuous variables were calculated and standardized [27]. Principal component analysis with varimax rotation was used to determine the internal structure of the predictor variables educational innovation, attention to competencies and learning environment. Following recommendations of Shönrock-Adema et al. [28] the internal structure has to be determined based on a combination of psychometric criteria and an investigation of the interpretability of possible factor solutions. Accordingly, factors were retained based on: (1) point of inflexion displayed by the scree plot, (2) eigenvalues > 1.5, (3) additional factors explain more than 5 % variance. The interpretability was assessed according to the following criteria: (4a) item loadings > 0.40, (4b) items of components share conceptual meaning, (4c) items on different components appear to measure different constructs, (4d) items load high on only one factor. The reliability of the scales was assessed using Cronbach’s alpha, with > 0.70 being considered adequate. Although Cronbach’s alpha is a common method to assess internal consistency, the measure is very sensitive to the number of items and tends to increase with scale length [29]. To examine the internal consistency of preparedness for practice (91 items), we also calculated Cronbach’s ρ which is an estimate of the mean inter-item correlation and is independent of scale length [30]. Values of rho vary widely and recommended values depend on the specificity of the construct under study. Minimal values between 0.15 and 0.20 are recommended for broad constructs [31]. Multicollinearity was assessed by inspection of the variance inflation factors and tolerance statistics [32]. In addition to basic descriptive statistics, correlation analysis and t-tests to compare means, stepwise hierarchical regression analysis was performed to examine the predictive values of the independent variables. To examine the direction of the proposed interaction between learning environment and attention to competencies, simple slope analysis was conducted [27].

A mediation or indirect effect is found when an independent variable influences a dependent variable through an intervening variable [33]. We analyzed the mediating effect of attention to competencies in the relationship between educational innovations and preparedness for practice (Fig. 1). The ‘indirect’ procedure as described by Preacher and Hayes [34] was used to calculate the 95 % confidence interval of the indirect effect. Assuming this mediation is supported and given the expected interaction between learning environment and attention to competencies, it is reasonable that the strength of the mediation also depends on the learning environment value. Accordingly, we used the ‘Modmed’ procedure [35], to test the significance of the mediation at different learning environment values.

## Results

Of the 330 questionnaires distributed, 143 complete questionnaires were returned (response 43 %), 84 (59 %) of which were completed by women, which reflects the gender distribution in undergraduate medical schools and PGME programmes in the Netherlands. The mean age was 39.7 (SD 4.7), and respondents had been licensed consultants for a mean of 4.2 (SD 2.3) years. Seventy-seven (54 %) were medical specialists, 36 (25 %) surgical specialists and 25 (18 %) supportive specialists, which is broadly consistent with outflow distributions of PGME programmes in the northeast educational region (59, 20 and 22 % respectively). Five respondents (4 %) did not report their speciality.

A principal component analysis (PCA) was conducted on the 6 items of educational innovations. The Kaiser-Meyer-Olkin measure (KMO = 0.77) showed sufficient sampling adequacy for PCA [31]. Moreover Bartlett’s test of sphericity χ^2^ (15) = 236.33, *p* < 0.001, indicated that correlations between items were sufficiently large for PCA. An initial analysis was run to obtain eigenvalues. Only 1 factor had an eigenvalue (2.9) above the cut-off score and explained 48 % of the variance. An inspection of the scree plot confirmed the 1 factor solution with the point of inflexion at the second factor. All items showed sufficiently large factor loadings (> 0.4). and the 1 factor solution complied with our interpretability criteria. Internal reliability was adequate with a Cronbach’s α of 0.78.

Using the same procedure PCA was run on the 7 items of the attention to competencies scale. KMO (0.82) and Bartlett’s test (χ^2^ (21) = 521.82, *p* < 0.001) showed adequate sampling adequacy and sphericity. One factor had an eigenvalue (3.9) above the threshold of 1.5 and explained 56 % of the variance. Inspection of the scree plot and application of the interpretability criteria confirmed the 1 factor solution. Internal reliability was adequate with a Cronbach’s α of 0.86.

A final PCA was conducted on the learning environment. KMO (0.81) and Bartlett’s test (χ^2^ (66) = 758.31, *p* < 0.001) showed adequate sampling adequacy and sphericity. Two factors had an eigenvalue (3.9 and 1.7) above the threshold of 1.5 and in combination explained 54 % of the variance. The scree plot was slightly ambiguous and showed inflexions that would justify a solution up to 3 factors. Varimax rotation was used to investigate solutions up to 3 factors. However, interpretability criteria were insufficient to differentiate unambiguously interpretable factors. Because the eigenvalue of the second factor was only slightly above the desired threshold, the 2 and 3 factor solutions were ambiguously interpretable and the 1 factor solution showed high reliability (Cronbach’s α of 0.85), we decided to treat learning environment as a single factor variable in further analyses.

The scale length independent mean inter-item correlation of preparedness for practice was adequate with a Cronbach’s ρ of 0.21. Men (M = 3.86) scored significantly higher on preparedness for practice than women (M = 3.69) (*p* = 0.03); therefore, gender was included in further analyses as a control variable. The outcome variable of preparedness for practice correlated significantly with all independent variables, most strongly with learning environment and attention to competencies (Table 2). No evidence of multicollinearity was found.

**Tab. 2:** Descriptives, Cronbach’s α and correlations

				Cronbach	Correlation				
		Mean	SD	α	1	2	3	4	5
1	Gender								
2	General self-efficacy	2.97	0.25	0.77	0.28**				
3	Educational innovations	3.03	0.82	0.78	0.05	0.01			
4	Attention to competencies	3.58	0.63	0.86	0.12	0.11	0.55**		
5	Learning environment	3.73	0.50	0.85	0.22**	0.21**	0.51**	0.71**	
6	Preparedness for practice	3.77	0.45	0.96	0.19*	0.36**	0.34**	0.58**	0.65**

*p < 0.05, **p < 0.01.

Hierarchical stepwise multiple regression analyses revealed the relative contribution of the independent variables on preparedness for practice (Table 3). First, only the control variables (gender and general self-efficacy) were entered: this model explained 14 % of the variance in preparedness for practice. General self-efficacy was significantly related to preparedness for practice but gender was not. The addition of educational innovations (second model) significantly increased the explained variance in preparedness for practice to 25 %. When attention to competencies was added to the model, this variable was significantly related to preparedness for practice, but educational innovations stopped being so (Table 3). This third model significantly increased the explained variance in preparedness for practice to 43 %. Because we expected an interaction between learning environment and attention to competencies, learning environment and the interaction term were added in the final model, which significantly increased the explained variance to 52 %. In this final model, general self-efficacy, attention to competencies, learning environment and the interaction term were significantly related to preparedness for practice, with learning environment as the strongest predictor.

**Tab. 3:** Summary of hierarchical regression analysis for variables predicting preparedness for practice (*n* = 143)

	Step 1		Step 2		Step 3		Step 4	
Variable	B	SE B	B	SE B	B	SE B	B	SE B
Gender	0.09	0.04	0.07	0.04	0.04	0.03	0.01	0.03
General self-efficacy	0.34**	0.04	0.35**	0.04	0.29**	0.03	0.23**	0.03
Educational innovations			0.33**	0.03	0.05	0.04	− 0.03	0.03
Attention to competencies (AtC)					0.51**	0.04	0.29**	0.04
Learning environment (LE)							0.42**	0.04
LE x AtC							0.13*	0.02
R^2^		0.14		0.25		0.43		0.52
R^2^ Change		0.14		0.11		0.18		0.09
F in R^2^ Change		11.19		19.88		43.06		12.7

**Step 1**: Model only including control variables.

**Step 2**: Effect of educational innovations, controlling for gender and self-efficacy.

**Step 3**: Additional effect of attention to competencies.

**Step 4**: Adding learning environment and interaction between LE and AtC.

All variables were standardized in advance, * *p* < 0.05, ** *p* < 0.01.

The significant interaction indicates that the relationship between attention to competencies and preparedness for practice varies by learning environment values. The simple slope for learning environment at 1 SD above the mean was significantly positive (B = 0.17, *p* < 0.001), but was only of borderline significance for a learning environment 1 SD below the mean (B = 0.09, *p* = 0.05). This means that attention to competencies was more strongly related to preparedness for practice for high learning environment values.

**Fig. 2:**
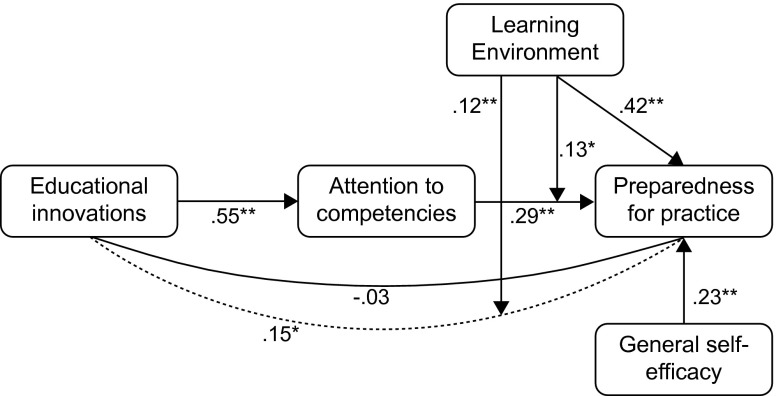
Model explaining preparedness for practice. *Dotted line* represents indirect effect. All values are standardized regression weights. *p < 0.05 ** *p* < 0.01

The 95 % confidence interval of the indirect effect between educational innovations and preparedness for practice through attention to competencies did not contain zero [0.10–0.21], which means that attention to competencies mediates the relationship between educational innovations and preparedness for practice. To examine the learning environment conditions under which this mediation was significant, we calculated 95 % confidence intervals of the indirect effect, 1 SD above and below the learning environment mean. The confidence interval was of borderline significance at 1 SD below the mean (0.00–0.13), and significant 1 SD above the mean (0.05–0.19), indicating that the indirect relationship between educational innovations and preparedness for practice through attention to competencies weakened as learning environment values decreased. An overview of the results is displayed in Fig. 2.

## Discussion

This study shows that the quality of the learning environment is of key importance in determining the degree to which novel competency-based PGME programmes prepare new consultants for independent practice. Although the educational innovations of competency-based PGME programmes were positively associated with preparedness for practice, this relationship was mediated by attention to competencies during feedback and coaching, and was no longer significant after adjusting for attention to competencies. In addition, this relationship varied by learning environment value, being strongest in a highly rated learning environment.

The application of educational innovations, such as portfolios or mini-CEX, was related to increased attention to competencies during feedback and coaching, which supports current directions in PGME [2]. A significant part of the educational innovations are assessment tools, and the use of these instruments was related to higher scores on preparedness for practice. Therefore, the indirect link between educational innovations and preparedness for practice through attention to competencies fits well with the claim that assessment drives learning [36]. However, subsequent analyses showed that the relationship between educational innovations and preparedness for practice was indirect, and mediated by attention to competencies. Because more time in PGME is spent on working independently in patient care than on feedback and deliberate education, this finding is not surprising, and supports earlier explanations of why the effects of curricular changes are difficult to measure directly [6, 8]. The indirect relationship suggests, furthermore, that although the educational innovations are valuable as a means to enhance attention to competencies, they should not become goals in themselves. Additionally, the fact that the regression coefficient of educational innovations in the final regression model was close to zero may suggest that strong programmes were more likely to have embraced innovative training methods. The indirect link between educational innovations and preparedness for practice may therefore not be a pure causal association and more research is needed to address this issue.

Educational innovations and attention to competencies seem to influence preparedness for practice, but the learning environment was found to be the strongest predictor. This finding is in line with claims that the learning environment has a unique contribution to the prediction of achievement, satisfaction and success in medical education [37, 38]. According to Genn [39], the learning environment forms the centre of educational change. Likewise, in our study, attention to competencies was not related to preparedness for practice when the learning environment scored low. This suggests that the current investments to change to competency frameworks will only pay off in terms of preparedness for practice when the learning environment is sufficiently well organized. However, how the learning environment in PGME influences learning is an under-researched area. The self-determination theory may be a promising theory to explain the relation between learning environment and learning [40]. According to this theory, the fulfilment of three innate needs (need for autonomy, need for competence and need for relatedness) is related to a range of outcomes such as better learning, better conceptual understanding, better academic achievement and higher levels of wellbeing [41]. A negative learning environment could limit self-motivation by offering less support for autonomy, relatedness and competence, which could ultimately result in new consultants feeling less well prepared for practice. However, more research is needed to fully understand these dynamics.

We acknowledge the following limitations to our study. First, the response rate was moderate, but common for physician surveys [42]. Although this may induce non-response bias, this is unlikely to distort the results meaningfully if the sample is representative [43], which was the case in our study. Moreover, the sample studied was large enough to allow valid statistical analyses. Second, all our data were obtained from self-report measures and common method variance could consequently be partly accountable for the relationships between the research model constructs [44]. However, as each individual uniquely experiences a training programme, we deliberately preferred self-report measures over objective measures that were only available at group level and therefore inadequate to do justice to the large variety in individual experiences. With respect to preparedness for practice, respondents were specifically asked to rate their training programmes to avoid common problems associated with self-assessment. And more importantly, general self-efficacy was included to control for the effect of self-efficacious beliefs on perceived preparedness for practice ratings. As a final limitation, the study was correlational, and although correlation is a necessary condition to make causal inferences, it is not sufficient to make such a claim with solid confidence.

A major strength of our study was the deliberate attempt to model various factors that influence preparedness for practice, to yield further insight into the effects of recent changes in PGME. Clarification studies using a similar approach are scarce in medical education and have recently been called for [45]. We feel that interventions to improve the connection between PGME and independent practice can improve as a result of a better understanding of the characteristics of PGME that contribute to new consultants’ feelings of preparedness for practice. Our study gives rise to several new research questions. Although more than half of the variance in preparedness for practice was explained by the variables included, a considerable part remained unexplained. More research is required to discover why some new consultants feel better prepared for practice than others and how training programmes can improve this connection. For example, with educational innovations and attention to competencies during coaching and feedback we included defining but not all aspects of CBE. More research is therefore needed to study the influence of other features of competency-based training as, for example, the variable advancement in training programmes. Furthermore, the significant influence of general self-efficacy on preparedness for practice suggests that the personal characteristics of trainees are important in determining the outcome of PGME programmes. More research is therefore needed to examine the importance of other personal characteristics. And although preparedness for practice is a useful and important outcome measure for PGME, other possible outcome measures such as job satisfaction, engagement, peer judgment or patient outcomes require further study.

In conclusion, our study showed that educational innovations and attention to competencies in PGME were related to higher levels of preparedness for practice, most strongly in a supportive learning environment. The success of PGME programmes, therefore, seems largely dependent on the degree to which a strong learning environment can be achieved.

### Essentials

- It is important to invest in the learning climate to prepare trainees optimally for independent practice.
- The effectiveness of didactic aspects of PGME seems to benefit from a positive learning environment.
- There is a positive relation between attention to competencies during coaching and feedback and preparedness for practice, which supports competency-based education.

#### Declaration of interest

The authors report no declarations of interest.
